# Acute enteritis: dehydration and rehydration in pediatric patients

**DOI:** 10.1186/1824-7288-40-S1-A59

**Published:** 2014-08-11

**Authors:** Piera Dones

**Affiliations:** 1 Pediatric Infective Diseases U.O.C., G. Di Cristina Hospital, Palermo, Italy

## 

Acute infectious enteritises are widespread globally. In developing countries, they are one of the leading causes of infant mortality. In developed countries, they account for a high number of outpatient visits and hospitalizations, with an economic impact on national health systems and, considering indirect costs, on communities. In roughly 70% of cases, the infections are caused by viral agents, principally rotavirus. A study conducted in collaboration with the Regional Referral Center for the Monitoring of Rotavirus Infection found that the serotypes isolated in all rotovirus-positive samples in the province of Palermo were 68% G1P8, and in 12.9% G2P4. In 20% of the cases, infectious gastroenteritis was caused by bacterial agents. At our hospital, the prevalent agents are salmonella spp. and campylobacter. For viral infections, there are no epidemiologic differences between developed countries and developing countries, though in the latter there is a higher mortality rate, associated with more difficult access to health care, malnutrition, and underlying diseases. Rehydration is first line treatment for pediatric enteritis. Indications for parenteral rehydration are generally quite restricted, and reserved for cases of severe dehydration, or cases in which oral rehydration solutions cannot be given either because of uncontrollable vomiting or because they cannot sufficiently restore electrolytes. A recent meta-analysis published in *Pediatrics* found that hypotonic pediatric solutions are the most widely used in hospitalized children. Use of these low-sodium solutions can lead to hyponatremia. Therefore, pediatric patients should always be rehydrated with isotonic solutions (0.9% NSS). For patients with hyponatremia, treatment should be slowly extended over a period of 24 hours, and sodium gradually supplemented in relation to total body water in order to avoid pontine myelinolysis, which is most frequent in dehydrated patients with severe underlying disease. However, it has also been found in otherwise healthy patients in whom treatment of hyponatremia was administered too rapidly. In fact, too rapid treatment can also lead to cerebral edema and/or seizures. Treatment should, in fact, be administered gradually over a period of 48-72 hours. Hypernatremic patients with hypovolemia clear more free water than solutes, so the total concentration of corporeal sodium is likely lower than that found in testing; therefore, treatment should also include isotonic fluids.

